# Imaging of Primary Liver Carcinosarcoma Scintigraphically; A Case Report

**DOI:** 10.4274/Mirt.260

**Published:** 2014-02-05

**Authors:** Ümmühan Abdulrezzak, Mustafa Kula, Zeynep Erdoğan, Ahmet Tutuş

**Affiliations:** 1 Erciyes University School of Medicine, Department of Nuclear Medicine, Kayseri, Turkey

**Keywords:** liver neoplasms, scintigraphy, bone formation

## Abstract

Primary liver carcinosarcoma is a very rare disease. There have been only a few cases described to date, none of which has been imaged by bone scintigraphy. A 69-year-old man who developed right back pain and weight loss was admitted to our hospital. Tenderness of the right upper abdomen, hepatomegaly, and a giant mass were the main physical examination signs. Abdominal ultrasonography showed a large lobulated heterogeneous echogenic solid mass with multiple cystic areas of varying size and a highly echogenic region that had posterior acoustic shadowing within the mass in the right lobe of the liver. Labeled erythrocyte imaging and ^99m^Tc-nanocolloid liver-spleen scan were performed to exclude hemangioma, showing a large intrahepatic photon deficient area. ^99m^Tc-methylene diphosphonate (MDP) bone scintigraphy revealed heterogeneous, irregular and dense activity accumulation thought to be osteoblastic activity in the liver mass. Postsurgical pathological diagnosis revealed “carcinosarcoma”.

**Conflict of interest:**None declared.

## Abstract (tr)

Primer Karaciğer Karsinosarkomu çok nadir bir hastalıktır. Bu güne kadar yalnızca birkaç vaka yayınlanmış olup, hiçbiri kemik sintigrafisinde görüntülenmemiştir. Hastanemize 69 yaşında hasta sırt ağrısı ve kilo kaybı şikayeti ile başvurdu. Fizik muayenede sağ üst batında hassasiyet, hepatomegali ve dev bir kitle tespit edildi. Abdominal USG’de içersinde multipl kistik alanlar içeren büyük lobüle heterojen ekojenik solid kitle ve kitle içinde posterior akustik gölge oluşturan yüksek ekojen alan izlenmekteydi. Hemanjiyomu dışlamak için yapılan eritrosit işaretleme çalışması ve Tc-99m nanookolloid sintigrafisi, karaciğer içinde aktivite tutulumu olmayan geniş bir alan göstermekteydi. Tc-99m Metilen Difosfonat Sintigrafisinde, karaciğerde osteoblastik aktivite ile uyumlu düzensiz yoğun aktivite akümülasyonu gösteren kitle lezyonu izlenmekteydi. Cerrahi patolojik tanı “karsinosarkom” olarak raporlandı.

## INTRODUCTION

Primary liver carcinosarcoma is a very rare disease. They represent less than 1% of primary malignant tumors of the liver (1). There have been only a few cases described to date but, none of which has been imaged by bone scintigraphy (1,2,3,4,5,6). Here we present a case, with an interesting appearance on bone scan.

## CASE REPORT

A 69-year-old man who developed right back pain and lost weight (12 kg in the last 2 month) was admitted to our hospital. Tenderness of the right upper abdomen, hepatomegaly, and a giant mass were the main physical examination signs. Serum markers for hepatitis B was positive, but the patient had no evidence of cirrhosis. He had moderately elevated bilirubin levels (total bilirubin 1.46 mg/dl (0.3-1.2 mg/dl), direct bilirubin 0.35 mg/dl (0-0.2 mg/dl)) and the other biochemical and hepatic function tests were as follows: erythrocyte sedimentation rate 103 mm/h, AST 65 U/l (0-35 U/l), ALT 34 U/l (0-45 U/l), alkaline phosphatase 382 U/l (30-120). The tumor markers revealed negative results: alpha-fetoprotein 2.7 ng/mL (<9.6 ng/mL); carcinoembryonic antigen 1.5 ng/mL (<5.0 ng/mL); serum carbohydrate antigen 19.9 10.97 U/mL (<39 U/mL).

Abdominal ultrasonography showed a large lobulated heterogeneous echogenic solid mass with multiple cystic areas of varying size and a highly echogenic lesion that had posterior acoustic shadowing within the mass in the right lobe of the liver.

**Figure 1:**
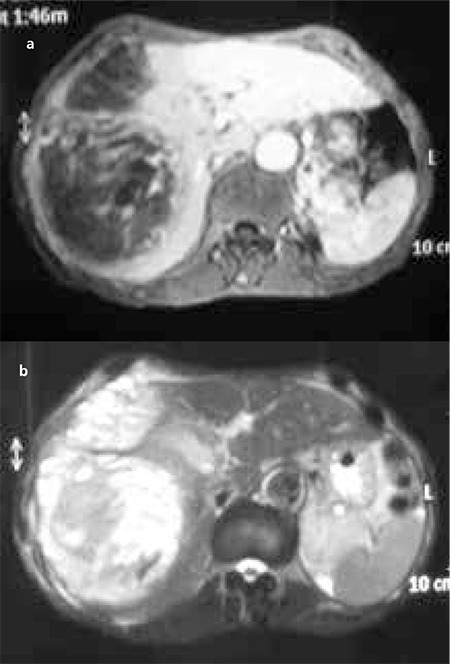
Magnetic resonance imaging. There was a large mass (152x120x135 mm) with heterogeneously hypointense on the T1-weighted image **(a)**, and heterogeneously hyperintense on the T2-weighted image. After the administration of contrast material, heterogeneous hyperenhancement was observed in the solid parts of the lesion **(b)**. This mass covered segment 7-8 completely and segment 4 partially.

On magnetic resonance imaging, this mass covered segments 7-8 completely and segment 4 partially and observed to be 152x120x135 mm in diameter. It was heterogeneously hypointense on the T1-weighted image (Figure 1a) and heterogeneously hyperintense on the T2-weighted image. After the administration of contrast material, heterogeneous hyperenhancement was observed in the solid parts of the lesion (Figure 1b).

**Figure 2:**
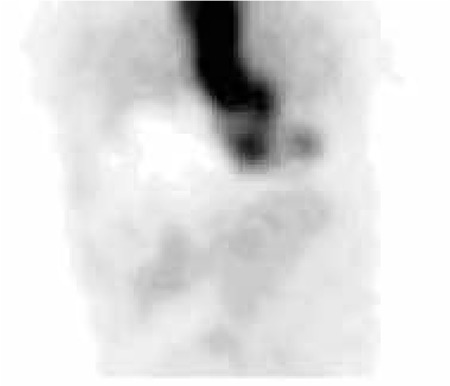
Labeled erythrocyte scan. The coronal slice showed a large cold area in the right lobe of the liver similar to the nanocolloid scan.

**Figure 3:**
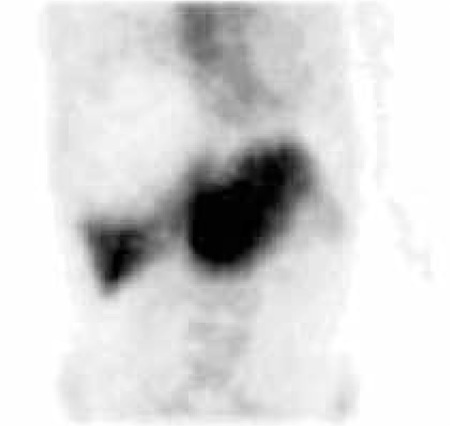
Tc-99m nanocolloid liver scan. The anterior view showed a large cold area replacing most of the right lobe, leaving a rim of normal right lobe uptake and a larger left lobe.

According to these imaging findings, the hepatic mass was suspected to be a hemangioma. Therefore, labeled erythrocyte imaging (Figure 2) and ^99m^Tc-nanocolloid liver-spleen scan (Figure 3) were performed to validate hemangioma diagnosis. The scintigraphic finding of a large intrahepatic photon deficient area was not suggestive of hemangioma.

**Figure 4:**
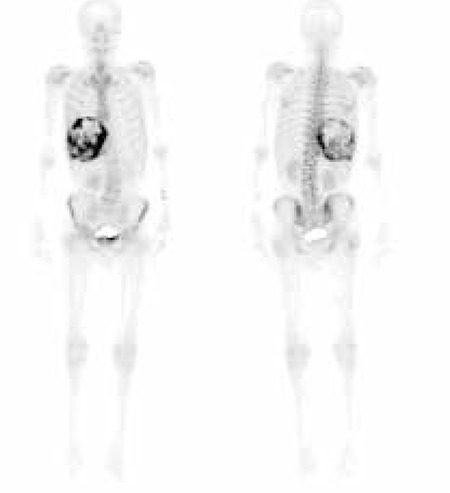
Whole-body bone scan with ^99m^Tc MDP showed intense irregular uptake in the right-sided liver mass. There wasn’t any pathological uptake in the bone structures.

Subsequently a ^99m^Tc-MDP whole-body bone scintigraphy was performed to rule out metastatic disease related to suspected primary malignant liver lesion. After the intravenous injection of 740 MBq (20 mCi) of ^99m^Tc-MDP, the patient was imaged on a dual-head coincidence camera (Siemens) with a low-energy high resolution (LEHR) collimator attached. Standardized anterior and posterior whole-body images were obtained. Images were acquired on a 256x1024 matrix using a scan speed of 12 cm/min. This study (Figure 4) revealed avid and heterogeneous uptake in the liver mass. Apart from this finding, the bone structures were normal. Postsurgical pathological diagnosis of the hepatic mass revealed carcinosarcoma (malignant mixed epithelial and stromal tumor). Histologically, the tumor showed an undifferentiated spindle cell neoplasm with foci of osteoid formation.

## LITERATURE REVIEW AND DISCUSSION

Carcinosarcoma is a malignant tumor that is a mixture of carcinomatous and sarcomatous elements, which most commonly arises from the ovary, uterus, and urinary bladder (7). Primary liver carcinosarcoma is a rare malignant hepatic tumor. Liver carcinosarcoma occurs most commonly in men between 50 and 70 years old. Some tumor markers may be abnormal, but the AFP level is usually normal (8,9).

In establishing the diagnosis of liver carcinosarcoma, it is important to demonstrate first that the tumor is histologically characteristic of carcinomatous (either hepatocellular or cholangiocellular) and sarcomatous elements, including malignant mixed tumors. This neoplasm consists of increased cellular pleomorphism, spindle cell stroma with abundant mitoses, osteogenesis, and atypical osteoid formation (13).

On MRI, hepatic carcinosarcoma is hypointense on the T1-weighted image and heterogeneously hyperintense on the T2-weighted image. Calcification and ossification, which are better seen on CT, have low or dark signal intensity on T1- and T2-weighted images (6,9).

A literature review showed that there are few reported cases of this type of primary hepatic tumor (3,4,5,6,7,10,11,12). Our patient had intense heterogeneous accumulation of a bone agent in his large liver mass but no noticeably pathological uptake had been identified at another site.

While ^99m^Tc MDP bone scans are primarily used to assess skeletal pathology, many other non-osseous lesions may accumulate bone seeking radiopharmaceuticals. These are often identified incidentally, while assessing underlying skeletal abnormalities. Extra osseous uptake of bone-seeking radiopharmaceuticals is seen in a wide variety of pathologic processes involving almost any organ. Although some possible mechanisms were proposed, soft tissue uptake of phosphate compounds such as diphosphonates is not well understood. Cause of extraosseous uptake of bone seeking radiopharmaceuticals may be related to ion exchange between intracellular calcium phosphates, increased calcium content of the tissue and the possibility of high concentration of phosphatase enzyme systems in certain tumors (14).

Hepatic uptake of ^99m^Tc diphosphonates is an unusual finding in nuclear medicine practice. There is a need for awareness of the pathophysiologic basis underlying such uptake, as it may be of critical clinical relevance in the evaluation of the patient. Localized areas of increased tracer uptake in the hepatic parenchyma are usually due to primary hepatic carcinoma or liver metastases.

In conclusion, the scintigraphic findings of hepatic carcinosarcoma presented in this case showed a large mass with irregular, dense activity accumulation thought to be osteoblastic activities. Especially if localized dense activity accumulation giving a mass image is seen in bone scan, carcinosarcoma should be included in the differential diagnosis of a large hepatic mass.
